# Warming and eutrophication interactively drive changes in the methane-oxidizing community of shallow lakes

**DOI:** 10.1038/s43705-021-00026-y

**Published:** 2021-07-05

**Authors:** Thomas P. A. Nijman, Thomas A. Davidson, Stefan T. J. Weideveld, Joachim Audet, Chiara Esposito, Eti E. Levi, Adrian Ho, Leon P. M. Lamers, Erik Jeppesen, Annelies J. Veraart

**Affiliations:** 1 Department of Aquatic Ecology and Environmental Biology, Institute for Water and Wetland Research, Radboud University, Nijmegen, The Netherlands; 2 Lake Group, Department of Bioscience and Arctic Research Centre, Aarhus University, Silkeborg, Denmark; 3 WATEC Aarhus University Centre for Water Technology, Aarhus University, Silkeborg, Denmark; 4 Institute for Microbiology, Liebniz Universität Hannover, Hannover, Germany; 5 Sino-Danish Centre for Education and Research (SDC), University of Chinese Academy of Sciences, Beijing, China; 6 Limnology Laboratory, Department of Biological Sciences and Centre for Ecosystem Research and implementation, Middle East Technical University, Ankara, Turkey; 7 Institute of Marine Sciences, Middle East Technical University, Erdemli-Mersin, Turkey

**Keywords:** Climate-change ecology, Community ecology, Freshwater ecology, Biogeochemistry, Microbial ecology

## Abstract

Freshwater ecosystems are the largest natural source of the greenhouse gas methane (CH_4_), with shallow lakes a particular hot spot. Eutrophication and warming generally increase lake CH_4_ emissions but their impacts on the sole biological methane sink—methane oxidation—and methane-oxidizer community dynamics are poorly understood. We used the world’s longest-running freshwater climate-change mesocosm experiment to determine how methane-oxidizing bacterial (MOB) abundance and composition, and methane oxidation potential in the sediment respond to eutrophication, short-term nitrogen addition and warming. After nitrogen addition, MOB abundance and methane oxidation potential increased, while warming increased MOB abundance without altering methane oxidation potential. MOB community composition was driven by both temperature and nutrient availability. Eutrophication increased relative abundance of type I MOB *Methyloparacoccus*. Warming favoured type II MOB *Methylocystis* over type I MOB *Methylomonadaceae*, shifting the MOB community from type I dominance to type I and II co-dominance, thereby altering MOB community traits involved in growth and stress-responses. This shift to slower-growing MOB may explain why higher MOB abundance in warmed mesocosms did not coincide with higher methane oxidation potential. Overall, we show that eutrophication and warming differentially change the MOB community, resulting in an altered ability to mitigate CH_4_ emissions from shallow lakes.

Freshwater ecosystems are globally the largest source of natural emissions of the greenhouse gas methane (CH_4_).^1^ They account for 43% (159 Tg CH_4_ year^-1^) of total natural CH_4_ emissions, of which about 70% originates from lakes,^1^ especially shallow lakes.^2^ Currently, many freshwater ecosystems are changing due to a combination of climate warming and increased phosphorus and nitrogen loading,^3^ which is expected to increase shallow lake CH_4_ emissions.^4,5^ In shallow lakes, methanogenic archaea produce CH_4_ predominantly in anoxic sediments. Subsequently, up to 90% of this CH_4_ is oxidized by methanotrophic microorganisms,^6^ mostly methane-oxidizing bacteria (MOB), which are abundant in the oxic top layer of the sediment. Therefore, MOB are key in mitigating CH_4_ emissions from freshwaters.

While it is known that eutrophication and warming lead to increased CH_4_ emissions,^4,5^ we lack a mechanistic understanding of the response of MOB communities to these stressors. Limitation and excess of nitrogen (N) and phosphorus (P) may significantly affect MOB activity depending on species-specific traits,^7,8^ while rising temperatures have been found to enhance methane oxidation potential.^9^ However, we do not yet know their combined effect on methane oxidation, and we particularly lack insight into the changes in MOB community composition as a result of these disturbances. Since MOB communities comprise a diverse set of microorganisms possessing different functional traits and roles,^10^ understanding the changes in the MOB community composition will help to understand the dynamics of methane oxidation in lake sediments.

The two largest groups of aerobic MOB are type I (Gammaproteobacteria) and type II (Alphaproteobacteria) MOB.^11^ Several studies have shown that type I and II MOB occupy different niches. While type I MOB are generally favoured by high methane and nutrient availability,^10^ type II have been found to be more resilient to disturbance and thrive under oligotrophic conditions.^12,13^ However, these characteristics were not explicitly linked to functioning of the community, most importantly methane oxidation potential. How the relative abundance of type I and II MOB affects methane oxidation potential will shed new light on the mitigation capacity of MOB communities. Here, we aim to identify how MOB communities change in response to eutrophication and warming and how that impacts the methane oxidation potential of freshwater sediments.

We sampled the sediment of a long-running lake mesocosm experiment to test the combined and separate effect of temperature and nutrients on methane oxidation potential, MOB abundance and MOB community composition. The setup consisted of two nutrient treatments (N + P added or not added) and three temperature treatments (ambient, +2–3 °C and +4–5 °C.^14^) To test for specific effects of N-availability, the year before sampling no N was added, while weekly P-addition in the high nutrient treatments continued. We sampled the top 4 cm of the sediment four days before N-addition was resumed (week -1, June), two months after (week 8, August) and one year after (week 52, June). We measured methane oxidation potential in bottle incubations and estimated MOB abundance by qPCR targeting the *pmoA* gene using the A189F and mmb661R primers. While these primers omit some MOB, in particular Verrucomicrobia and NC10,^15^ they target the majority of proteobacterial MOB. MOB community composition was determined by 16S rRNA gene amplicon sequencing and analysed in the dada2 pipeline^16^ using the Silva v138 database^17^ to assign taxonomy. In addition, we calculated the type I:type II MOB ratio and measured diffusive and ebullitive CH_4_ fluxes and a range of physicochemical properties of the sediment and water column (Supplementary methods, Figs. S1–11).

MOB abundance was significantly affected by nutrients (*p* = 0.018), N-addition (*p* = 0.044) and temperature (*p* < 0.001, Fig. 1A, Table S1A, three-way mixed ANOVA). MOB abundance increased two months after resuming N-addition (*p* = 0.012), and was higher in warmed mesocosms than at ambient temperature (*p* < 0.0001 for both +2–3 °C and +4-5 °C, Table S1B). Methane oxidation potential was also affected by nutrients (*p* = 0.002) and N-availability (*p* < 0.0001) but not by temperature (*p* = 0.292, Fig. 1B, Table S2), and therefore did not follow the temperature-response of MOB abundance, leading to a lower apparent cell-specific activity with increased temperature (*p* < 0.0001, Fig. 2A, Table S3).

**Fig. 1:**
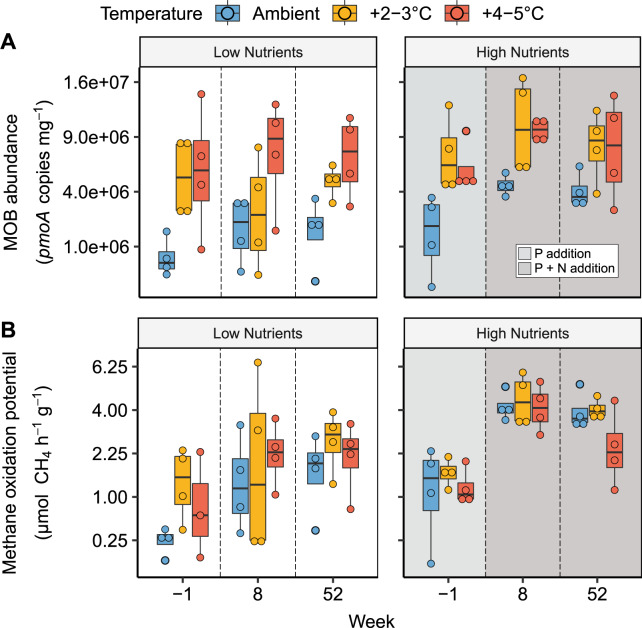
MOB abundance and methane oxidation potential in response to temperature and nutrients. MOB-abundance (*pmoA* gene copy number) (**A**) and Methane oxidation potential (**B**) (both square-root transformed). The setup consisted of 24 mesocosms with three temperature treatments (Ambient, +2–3 °C, + 4–5 °C) and two nutrient treatments (no N/P added, N/P added every 2 weeks). The year before sampling no N was added (apart from natural input with the groundwater added) while P-addition was continued. N-addition was resumed four days after the ‘week -1’ sampling. Boxes indicate the first and third quartiles, lines indicate the median, whiskers indicate outer data points if less than 1.5*interquartile range from quartiles. Colours represent temperature treatments. Individual data points are shown, *n* = 71.

**Fig. 2:**
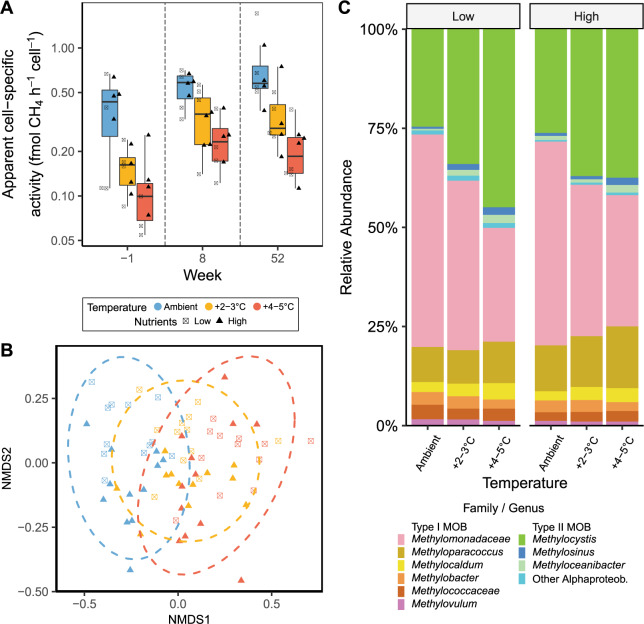
Apparent cell-specific activity and MOB community composition in response to treatments. **A** Apparent cell-specific methane uptake rate. The setup consisted of 24 mesocosms with three temperature treatments (Ambient, +2–3 °C, + 4–5 °C) and two nutrient treatments (no N/P added, N/P added every 2 weeks). The year before sampling no N was added (apart from natural input with the groundwater added) while P-addition was continued. N-addition was resumed 4 days after the ‘week -1’ sampling. High and low nutrient treatments were combined as nutrients did not significantly affect the apparent cell-specific activity. Boxes indicate the first and third quartiles, lines indicate the median, whiskers indicate outer data points if less than 1.5*interquartile range from quartiles. Colours represent temperature treatments. Triangles indicate high nutrient treatments, crossed squares low nutrient treatments, *n* = 71. **B** NMDS of MOB community. Colours represent temperature treatments. Triangles indicate high nutrient treatments, crossed squares low nutrient treatments. **C** Relative abundance of MOB with >1% abundance, ASVs grouped together based on the level they were identified, either at the Genus level or the Family level. Genera with <1% abundance were added to the ‘Other Alphaproteob.’, ‘*Methylomonadaceae*’ or ‘*Methylococacceae*’ groups, depending on their phylogeny. Different weeks are grouped together. Colours represent the different MOB Genera and Families.

NMDS analysis showed that MOB communities clustered based on temperature and nutrients (Fig. 2B). The fact that the community clustered more based on temperature and nutrients than sole N-addition, indicates that in this experiment community composition is likely mostly influenced by long-term processes. Temperature (*p* < 0.0001), nutrients (*p* = 0.001) and N-addition (*p* < 0.0001) significantly affected community composition, with temperature having the strongest effect, based on PERMANOVA analysis (Table S4A). Warmed treatments had a significantly different community composition from the ambient treatment in both nutrient treatments (Table S4B).

Eutrophication increased relative abundance of *Methyloparacoccus* (Fig. 2C, Table S5). Warming shifted MOB communities from type I dominance, in particular because of high relative abundance of type IA *Methylomonadaceae*, to type I and II co-dominance, mostly due to an increase in *Methylocystis*, decreasing the type I:type II ratio (*p* < 0.0001, Fig. S1, Tables S6, S7). This coincided with a decrease in apparent cell-specific activity (Fig. 2A), indicating a potential mechanistic effect.

A recent study showed that slow-growing microorganisms are more competitive at higher temperatures, and are expected to be favoured by global warming.^18^ Indeed, type II MOB generally exhibit a more oligotrophic, slow-growing life strategy than type I MOB,^10^ and are resistant to high temperatures,^13^ potentially explaining their increased relative abundance at higher temperature. Also, slower-growing oligotrophs have a higher yield per molecule of substrate and take up substrate at a lower rate than fast-growers.^19^ Thus, in a community dominated by slow-growing organisms, the total amount of substrate used per day per organism is lower than in a community dominated by fast-growers. This may explain why the shift to type II MOB coincided with lower apparent cell-specific activity, and why the increased MOB abundance in warmed mesocosms, which was mostly due to an increase in type II MOB, did not increase methane oxidation potential.

In contrast, N-addition increased methane oxidation potential. The effects of N have been found to be dose-dependent,^20^ N-substrate dependent,^8^ and species-dependent.^8^ N additions can either relieve MOB from N-limitation, or inhibit methane oxidation potential.^20^ Here, N-dependent stimulation of methane production, as shown by increased methane emissions (Fig. S2), likely fuelled the higher methane oxidation potential, in concert with relieved N-limitation of the MOB.

In conclusion, our results show important differential effects of eutrophication and warming on MOB communities. While warming shifts lake sediments from type I MOB dominance to type I and II MOB co-dominance without affecting overall community activity, methane oxidation potential is enhanced by eutrophication. To accurately predict effects of these MOB community shifts on CH_4_ emissions, future studies should also address interactive warming and eutrophication effects on methane producing communities.

## Supplementary information

Supplementary Information

## Data Availability

Experimental data are available at the Dryad Digital Repository (10.5061/dryad.djh9w0w01). Sequencing data are deposited at the European Nucleotide Archive under accession number PRJEB43466.

## References

[1] Saunois M (2020). The global methane budget 2000-2017. Earth Syst. Sci. Data.

[2] Bastviken D, Cole J, Pace M, Tranvik L (2004). Methane emissions from lakes: dependence of lake characteristics, two regional assessments, and a global estimate. Glob. Biogeochem. Cycles.

[3] Moss B (2011). Allied attack: climate change and eutrophication. Inl. Waters.

[4] Davidson TA (2018). Synergy between nutrients and warming enhances methane ebullition from experimental lakes. Nat. Clim. Chang..

[5] Aben RCH (2017). Cross continental increase in methane ebullition under climate change. Nat. Commun..

[6] Oremland RS, Culbertson CW (1992). Importance of methane-oxidizing bacteria in the methane budget as revealed by the use of a specific inhibitor. Nature.

[7] Veraart AJ, Steenbergh AK, Ho A, Kim SY, Bodelier PLE (2015). Beyond nitrogen: the importance of phosphorus for CH4 oxidation in soils and sediments. Geoderma.

[8] Hoefman S (2014). Niche differentiation in nitrogen metabolism among methanotrophs within an operational taxonomic unit. BMC Microbiol..

[9] Shelley F, Abdullahi F, Grey J, Trimmer M (2015). Microbial methane cycling in the bed of a chalk river: oxidation has the potential to match methanogenesis enhanced by warming. Freshw. Biol..

[10] Ho A (2013). Conceptualizing functional traits and ecological characteristics of methane-oxidizing bacteria as life strategies. Environ. Microbiol. Rep..

[11] Semrau JD, Dispirito AA, Yoon S (2010). Methanotrophs and copper. FEMS Microbiol. Rev..

[12] Kaupper T (2021). When the going gets tough: emergence of a complex methane-driven interaction network during recovery from desiccation-rewetting. Soil Biol. Biochem..

[13] Ho A, Frenzel P (2012). Heat stress and methane-oxidizing bacteria: effects on activity and population dynamics. Soil Biol. Biochem..

[14] Liboriussen L (2005). Global warming: design of a flow-through shallow lake mesocosm climate experiment. Limnol. Oceanogr. Methods.

[15] Ghashghavi M, Jetten MSM, Lüke C (2017). Survey of methanotrophic diversity in various ecosystems by degenerate methane monooxygenase gene primers. AMB Express.

[16] Callahan BJ (2016). DADA2: high-resolution sample inference from Illumina amplicon data. Nat. Methods.

[17] Quast C (2013). The SILVA ribosomal RNA gene database project: improved data processing and web-based tools. Nucleic Acids Res..

[18] Lax S, Abreu CI, Gore J (2020). Higher temperatures generically favour slower-growing bacterial species in multispecies communities. Nat. Ecol. Evol..

[19] Lipson DA (2015). The complex relationship between microbial growth rate and yield and its implications for ecosystem processes. Front. Microbiol..

[20] Bodelier PL, Laanbroek HJ (2004). Nitrogen as a regulatory factor of methane oxidation in soils and sediments. FEMS Microbiol. Ecol..

