# Supplementary material for: Warming and eutrophication interactively drive changes in the methane-oxidizing community of shallow lakes
Source: ISME Commun. 2021 Jul 5;1:32. doi: 10.1038/s43705-021-00026-y (PMC9723669; doi:10.1038/s43705-021-00026-y)
Supplement: Supplementary file 1 — Supplementary Information [file 43705_2021_26_MOESM1_ESM.docx]

# **Supplementary information**

**Warming and eutrophication interactively drive changes in the methane-oxidizing community of shallow lakes**

Thomas P. A. Nijman^1^, Thomas A. Davidson^2,3^, Stefan T. J. Weideveld^1^, Joachim Audet^2,3^, Chiara Esposito^2,3^, Eti E. Levi^2,3^, Adrian Ho^4^, Leon P.M. Lamers^1^, Erik Jeppesen^2,3,5,6,7^ & Annelies J. Veraart^1^

**Contents**

- Supplementary methods 2
- Supplementary results and discussion 9
- Supplementary figures 11
- Supplementary tables 17
- Supplementary references 22

## **Supplementary methods**

### *Mesocosm experimental setup*

The Aarhus University Lake Mesocosm Warming Experiment (LMWE) is the longest running freshwater climate-change mesocosm experiment worldwide, and has been running since August 2003 [1]. It is situated in Central Jutland, Denmark (56°14′ N, 9°31′ E). The setup consists of 24 flow-through mesocosms (diameter 1.9 m, water depth 1 m, retention time ~2.5 months), which are continuously mixed and fed with groundwater. Two nutrient treatments, and three temperature treatments are applied to the mesocosms, resulting in a total of six treatments with four replicates each. Nutrient treatments consist of ‘low’ and ‘high’ nutrients. The ‘low nutrient’ treatment receives no additional nutrients, while the ‘high nutrient’ treatment receives 7 mg P m^−2^ day^−1^ and 27.1 mg N m^−2^ day^−1^ in the form of Na_2_HPO_4_ and Ca(NO_3_)_2_, respectively. The temperature treatments consist of ‘ambient’, in which temperature is not controlled, and two warmed treatments, which are continuously warmed according to IPCC scenario A2 (+2-3°C) and IPCC scenario A2+50% (+4-5°C), using the ambient mesocosm as the temperature reference. A seasonal gradient is applied to closely mimic the IPCC scenarios [2]. Additionally, the year prior to our sampling campaign (June 2018 - June 2019), no N was added to the high nutrient mesocosms. We sampled four days before (week -1), two months after (week 8) and one year after (week 52) N-addition was resumed.

### *Mesocosm sediment sampling*

Sediment samples for microcosm incubations and molecular analyses were taken by inserting tubes (Ø6cm) into the sediment by hand. Sediment samples were taken opposite to the mixing-paddle, where the sediment was least disturbed. We then sliced off the top 4 cm, taking care not to disturb the sediment. Sediment samples were sieved and then immediately transported back to the lab. Surface water samples, to add to the microcosms, were taken immediately after taking the sediment samples. Samples were kept at 4 °C and microcosm incubations to examine methane oxidation potential were started within 24 hours. The sediment was mixed and before using it for the microcosms, a subsample was taken for molecular analysis and frozen at -20 °C.

### *Microcosm incubations*

The sediment was mixed manually, after which 5 grams of fresh sediment for every mesocosm and 20 ml of surface water from the respective mesocosm were added to a 120 ml serum bottle for microcosm incubations. For week 8, microcosm incubations were carried out in triplicate. The serum bottles were closed with airtight red butyl rubber stoppers and capped, after which 1% CH_4_ was added to the microcosms. The microcosms were incubated at 20 °C for 24 hours on a gyratory shaker at 150 rpm. CH_4_ concentration in the headspace was measured at approximately t=0h, 6h and 18h. In microcosms with a low methane oxidation rate, an additional measurement was performed after 24 hours.

For week -1, CH_4_ was measured by taking 6 ml of headspace using a gas-tight glass syringe (Hamilton) and injecting into 3 ml pre-vacuumed Labco exetainers, creating overpressure to prevent air intrusion. To replace the headspace volume in the serum bottles, 6 ml N_2_-gas was added. CH_4_ concentrations in the exetainers were measured by injecting 100 µl headspace sample into an HP 5890 GC equipped with a flame ionization detector and a Porapak Q column (80/200 mesh), taking care to remove overpressure from the syringe before injection. Five measurements were removed because of suspected leakage of the exetainers. Calculations were adjusted based on CH_4_ removed during sampling. For week 8 and 52, CH_4_ concentration in the headspace was measured by directly injecting 100 µl into an SRI 8610C GC (SRI Instruments, Earl St. Torrance, California, USA) equipped with a 3' x 1/8" Silica Gel Packed Column and a flame ionization detector (FID). One measurement was removed because of suspected blockage of the GC. At both GCs, certified CH_4_ standards were used for calibration and validation. Finally, the methane oxidation potential was calculated from the slope of the CH_4_ decrease in the microcosms and corrected for the water content of the sediments to obtain hourly rates per gr dry weight. One microcosm (from the low nutrient, +4-5°C treatment in week -1) was discarded because of the low r-squared (<0.8) of the slope.

### *Molecular analyses*

DNA was extracted from the sediment using the Powersoil kit (Qiagen), with minor modifications to optimize the DNA yield. Before starting the extraction, we removed beads from the powerbead tubes, added 0.5g of sediment and then centrifuged the samples for 30s at 10.000 x *g*. We then removed the liquid phase, as suggested by the Powersoil Handbook for samples with high water content, weighed the samples again, and re-added the beads to the tubes. In step 9 and 12 of the extraction, we transferred all the supernatant rather than part of it. The reason was that because of the high water content of some of the samples, there was a large variation in the total amount of supernatant. Taking only part of the supernatant would have affected later qPCR results. Furthermore, our samples did not show a high humic and fulvic acid content, and therefore we did not expect that taking a larger amount of supernatant would inhibit the efficiency of the DNA extraction. In the final step, we added 50 µl of solution C6 twice, waiting 2-3 minutes before centrifuging, similar to [3]. DNA concentrations were measured using a Qubit fluorometer (Invitrogen).

We measured *pmoA* gene copy number, a proxy for MOB abundance, by quantitative PCR (qPCR) using the primers A189F and mmb661R [4]. The cycle had 3 minutes denaturation at 95°C, 45 cycles of 10s 95°C, 15s 58°C, 25s 72°C and 10s 82°C, ending with a melt-curve in steps of 1°C from 70°C to 95°C [4]. DNA was diluted and 1 ng of DNA was added to all reactions. All samples and standards were measured in triplicate for every run. qPCR was performed on a BioRad iQ5 Multicolour Real-Time PCR detection system (Vers. 2.0, BioRad, Gothenburg Sweden), with iQ SYBR® Green Supermix as mastermix. Melt curves were checked on the BioRad iQ5, and copy number of the *pmoA* gene was calculated.

MOB community composition was determined by amplicon sequencing, which was performed by Novogene, on the Novaseq platform. The V3-V4 bacterial 16S region was sequenced using the universal 341F (CCTAYGGGRBGCASCAG) and 806R (GGACTACNNGGGTATCTAAT) primers [5]. We did two sequencing runs, one including all samples of week -1 and week 8, and one with all samples of week 52. Amplicon data of the two runs were processed separately and merged after assigning taxonomy. The dada2 pipeline was used to process the reads [6]. Because the overlap between the reads was too short for merging the reads, we used only the forward reads for our analysis (as described in the big data tutorial on <https://benjjneb.github.io/dada2/bigdata.html>). The *FilterAndTrim* function was used with parameters TruncLen=0, maxEE=1 and truncQ=10. Error rate was inferred using all reads, and adapted because of the Novaseq limitations (according to <https://github.com/benjjneb/dada2/issues/791>). The majority of reads had a length of 227 bp, reads of other lengths were excluded. Finally, chimeras were removed with the *removeBimeraDenovo* function. This resulted in a total of 6 232 628 reads in the first run and 2 702 136 reads in the second run. ASVs were determined and taxonomy was assigned using the v138 Silva database [7]. Reads per sample were then rarefied without replacement according to the lowest sample size of 64,434 reads to adjust for bias in sampling effort [8]. Data was analysed using the *phyloseq* [9] and *microbiome* [10] packages.

A subset was taken of all aerobic MOB ASVs, by including all bacteria that had “*Methylo*” in their family name. We initially found that the Silva database had assigned several ASVs to the *Crenothrix* genus. As *Crenothrix* is a relatively rare genus [11], and Oswald et al.(2017) showed that *Crenothrix* sequences are quite closely related to other type Ia MOB, we further checked the classification of the ASVs associated to *Crenothrix* that had an abundance over 0.5%. We found that in a BLAST nucleotide search [13], all of these sequences were instead related to *Methylobacter* (table S7). Furthermore, another check was performed by assigning taxonomy using the RDP training set 18 [14]. All *Crenothrix* sequences were assigned to *Methylobacter*, similar to the BLAST search. Considering these lines of evidence, we suspect that the the ASVs assigned to *Crenothrix* have been mis-classified at the genus level, based on the SILVA database. We now retain the classification to the preceding taxonomic level (*Methylomonadaceae*). `*Methylomonadaceae*` is the provisional family to accommodate type Ia methanotrophs, including *Methylobacter*, based on the 16S rRNA gene.

### *Sediment analyses*

Sediment samples of the top 4 cm, which were also used for the microcosm incubations, were used to determine available phosphorus, water content and organic matter content of the sediment. Plant and microorganism available phosphorus in the sediment were quantified using the P-Olsen extraction method [15]. P concentrations in the extractions were measured using inductively coupled plasma optical emission spectrometry with the iCAP 6000 (Thermo Fischer Scientific, Bremen, Germany). Water content was determined by drying the samples for 72 hours and organic matter content by heating the dried samples at 550 °C for 4 hours.

### *Surface water analysis*

We analysed a range of physicochemical variables in the surface water. We measured dissolved oxygen with an OxyGuard oxygen sensor and temperature with a PR electronics temperature sensor in the surface water of each mesocosm at 50 cm depth. Both variables were measured every 30 mins and the daily average value was used for our analysis. We measured pH directly after measuring CH_4_ diffusive flux with an Intellical PHC101 pH sensor attached to a Hach HQ40D Portable Multi Meter in the top of the mesocosms.

To determine total nitrogen, samples were taken in week -1, 8 and 52 in the high nutrient mesocosms and in week 8 and 52 in the low nutrient mesocosms. The samples, which were retrieved with a tube sampler from three different points in the mesocosms, were pooled and subsequently subsampled for water chemistry analysis. Total nitrogen, comprising free ammonia, ammonium, nitrite, nitrate, and organic nitrogen compounds, was analysed with a FIAStar 5000 according to the application note (AN5202) for the equipment by FOSS Analytics [16].

For chlorophyll *a* analysis, two litres of water were subsampled from the pooled water samples and were kept in dark and cold prior to extraction. Subsequently, water samples (100-900 ml) were filtered through Whatman GF/C filters and ethanol extraction was conducted according to Danish Standard 2201 [17]. Absorbance is measured with a Shimadzu UV-1800 spectrophotometer at wavelengths 665 and 750 nm. We lacked spectrophotometric chlorophyll *a* data for week -1 in the low nutrient tanks. These values were instead determined by chlorophyll *a* fluorescence data using the Turner Designs, USA, Cyclops-7f after calibrating against spectrophotometric measurements.

### *Porewater analysis*

Porewater samples were taken in week 8 and 52 to determine porewater CH_4_ concentrations. Samples were taken by carefully inserting ceramic porewater samplers in the sediment at 10 cm depth. In week -1, porewater samples were also taken, but at a deeper depth of approximately 25 cm. The results from week -1 were therefore excluded from the analysis. We took samples for CH_4_ porewater concentrations by attaching a vacuumed 12 ml Labco exetainer to the porewater sampler. The exetainers contained 1 ml 0.5 M HCl to immediately stop all microbial activity, and were filled halfway with porewater. We added N_2_-gas to the CH_4_ porewater samples to remove the vacuum and then equilibrated the CH_4_ in water and headspace by shaking and leaving the samples at room temperature for 30 minutes. 100 µl headspace sample was injected into a HP 5890 GC equipped with a flame ionization detector and a Porapak Q column (80/200 mesh) to quantify headspace CH_4_. CH_4_ concentrations in the porewater were calculated using Henry’s law.

### *Diffusive and ebullitive flux*

Diffusive fluxes of CH_4_ were measured in week -1, 8 and 52 with a transparent acrylic glass floating chamber (Ø30cm) connected to a Greenhouse Gas Analyzer (G2508 CRDS Analyzer, Picarro, Santa Clara, CA, USA). Each diffusive flux measurements lasting at least 300 second counted from when CH_4_ concentrations started to increase or decrease. When a sudden increase in CH_4_ concentration was observed (due to ebullition) the measurement was discarded and repeated. In between measurements the chamber was aerated in order to return to atmospheric CH_4_ concentrations. We used the linear change in gas concentration over time to calculate the diffusive flux of CH_4_ [18]_._

Ebullitive flux was captured in custom-designed bubble traps, consisting of two inverted funnels leading to 100 ml polypropylene syringes held in place by a rubber bung. The funnels have a 120 mm diameter and together cover an area of 0.0226 m^2^, which is 0.8% of the total area of the mesocosms. This is a relatively large proportion of the total area of the mesocosm when compared with the areas of real lakes samples in studies deemed to have produced reliable results [19]. The syringes were checked and sampled every two weeks from April to October and once a month during winter. The gas was sampled directly from the syringes connected to a three-way stopcock and then stored in 12 ml exetainer. The funnels contained a gauze to prevent animals entering the trap and the funnels, gauze and syringes were cleaned or replaced every two weeks over the course of the summer and every month in the winter. CH_4_ concentration in the samples was measured by directly injecting 30 µl into an SRI 8610C GC (SRI Instruments, Earl St. Torrance, California, USA) equipped with a 3' x 1/8" Silica Gel Packed Column and a flame ionization detector (FID). At both GCs, certified CH_4_ standards were used for calibration and validation.

Ebullitive flux of CH_4_ was estimated as:

$$\frac{{pCH4}_{gas}\times{Vol}_{bub}}{t\times A}$$

where ${pCH4}_{gas}$ is the concentration of CH_4_ in the gas that was trapped, ${Vol}_{bub}$ is the volume of gas trapped, $t$ is the time during which the bubble traps are in the mesocosms and $A$ is the area of the funnel (i.e., 0.226 m^2^). A fraction of the CH_4_ present in the bubble trap might have re-dissolved in the water in the syringes thus slightly underestimating the ebullitive flux. However, because of the low solubility of CH_4_, we assume this underestimation to be minor and correcting for it is unlikely to decrease the uncertainty in the data. A study [20] attempted to correct for this underestimation, assuming that the CH_4_ gas present in the bottle of the trap is in equilibrium with the CH_4_ dissolved in the water present in the bottle. As a result, their ebullitive flux increased by 2-5%.

### *Plant and algal cover*

Plant and filamentous algae surveys were conducted every other week during the study period, and percent coverage together with height (cm) were recorded. Percent volume inhabited were calculated based on coverage (%) ∗ plant height (cm)/water depth (cm) [21].

### *Statistical analysis*

MOB abundance and potential methane oxidation were square-root transformed to achieve normality. We used three-way mixed ANOVAs using the *rstatix* [22] package to test for differences between the treatments. Nutrients and temperature were included as between subject variables, while week (week numbers relative to resuming N-addition) was included as within subject variable. Pairwise Bonferroni-adjusted t-tests were used as post-hoc tests. Since there were no significant interactions, we tested post-hoc effects for main effects in the complete dataset. The only exception was sampling week, for which we performed post-hoc tests for the ‘high nutrient’ mesocosms only, since N-addition was only resumed in those mesocosms.

To calculate apparent cell-specific methane uptake rate, we first determined the average *pmoA* copy number of aerobic MOB. Therefore, we downloaded the available genomes of microorganisms with the *pmoA* gene frome MaGe [23]. We then excluded the sequences associated to Verrucomicrobia and NC10, as those are not targeted by the A189F and mmb661R primers [24] and calculated the average *pmoA* gene copies of the remaining MOB, which was 1.62. Last, we calculated the apparent cell-specific methane uptake rate by dividing the methane oxidation potential of the community by the *pmoA* gene copy number, then dividing by the average *pmoA* gene copy number of 1.62. Although other studies found weak correlations between MOB-abundance and activity, likely partly due to technical limitations of DNA-based quantification [17], in our study this discrepancy coincided with a change in community composition, indicating a potential mechanistic effect.

The ratio type I: type II MOB was calculated by dividing the number of reads of type I MOB by the number of reads of type II MOB. The specific methane oxidation rate and MOB ratio were log transformed to achieve normality. Three-way mixed ANOVAs were used to test for differences and interactions between treatments. Post-hoc tests were carried out in the same way as for MOB abundance and potential methane oxidation.

We used multivariate analysis to determine the effects of temperature, nutrients and N-addition on community composition of aerobic MOB, excluding MOB from the NC10 group. Before starting the analysis, data was chord transformed to reduce the effect of zeros on the analysis [25]. Non-metric dimensional scaling (NMDS), using the *vegan* package [26], was used to explore the variation in community composition. Because NMDS is a non-parametric form of ordination, and makes few assumptions about the nature of the data, it is suitable for a wide variety of datasets [27]. This is especially important for microbial datasets such as ours with many species of which some are very abundant. Using the *metaMDS* function, convergent solutions were found after 42 tries. Treatments were applied to the NMDS plot after the analysis.

Permutational MANOVA (PERMANOVA) [28] was used to test the effect of temperature, nutrients and N-addition on MOB community composition. PERMANOVA was chosen because it allows for testing the main effects and post-hoc effects of differences between groups, similar to ANOVA, but then for multivariate data with multiple response variables. We used PRIMER7 with the PERMANOVA+ add-on for our analysis [29]. PERMANOVA was tested on the Bray-Curtis dissimilarity matrix of MOB community composition. Mesocosm was added as random factor within the temperature and nutrient treatments to account for repeated measures. Post-hoc analysis of the effects of temperature on MOB community composition was also performed using PERMANOVA. Since there was a significant interaction between temperature and eutrophication, temperature treatments were compared for both low and high nutrient treatments. All tests were carried out with 99999 permutations. Monte-Carlo adjusted p-values were used to test significance.

Plots were made using the *ggplot2* [30] and *ggpubr* [31] packages. All analyses were performed in R version 3.6.3 [32].

## **Supplementary results and discussion**

Nutrient data clearly showed the difference between the high and low nutrient tanks. Total N in the surface water increased after resuming N-addition (figure S2). P-Olsen was much higher in the sediment of the high nutrient tanks at all times (figure S3). After resuming N-addition, P-Olsen increased, likely because of increased mineralization.

The diffusive and ebullitive CH_4_ flux increased after N-addition was resumed (figure S4). One year after resuming N-addition (so sampled in the same time of the year), fluxes were on average 14 times higher in the high nutrient mesocosms than before, clearly showing that the combination of N- and P-addition led to much higher emissions than only P addition. Also, the effect was much stronger than the seasonal effect, as there was a larger difference between the emissions of the first (week -1 and week 8) and the second (week 52) year than between those in June (week -1 and week 52) and August (week 8). Ebullition was the most important GHG pathway, contributing 87.8% of all CH_4_ emissions (91% of high nutrient mesocosm emissions and 72.5% of low nutrient mesocosm emissions). Most of these emissions came from the high nutrient mesocosms after resuming N-addition. In contrast to previous years [33], highest fluxes were found in the lower temperature treatments, both for ebullitive and diffusive flux (figure S4). This was likely due to the high chlorophyll *a* in the surface water of the lower temperature treatments (figure S5), and the presence of filamentous algae (figure S6) and macrophytes (figure S7) in the highest temperature tanks. Total N in the surface water showed a similar pattern as total flux (figure S2), with highest total N in low temperature mesocosms, decreasing with temperature. The higher amount of N was likely the cause of the higher chlorophyll *a* amount. In the low nutrient mesocosms there was no increase in CH_4_ flux, clearly showing the importance of N for CH_4_ emissions.

CH_4_ concentration in the porewater (figure S8) was influenced by eutrophication, but not by warming. CH_4_ concentration in the porewater was higher in the high nutrient mesocosms (F_1,18_=4.4, p=0.005), with on average 0.76 mmol CH_4_ L^-1^ in high nutrient mesocosms and 0.61 mmol CH_4_ L^-1^ in low nutrient mesocosms in week 8 and 52, a 26% increase. This increase was lower than the increase in methane oxidation potential in high nutrient mesocosms, which increased by 55% in high nutrient mesocosms (3.51 µmol h^-1^ g^-1^ at high and 2.27 µmol h^-1^ g^-1^ at low nutrients).

pH (figure S9) was influenced by long-term eutrophication (F_1,18_=7.6, p=0.013), but not by short-term nitrogen addition and warming, while oxygen concentration in the surface water (figure S10) was not influenced by any of the treatments. Although temperature (figure S11) showed a seasonal effect and was higher in weeks 8 and 52 after N-addition than in week -1, our data show that methane oxidation potential was affected by eutrophication rather than temperature.

Patterns in DNA are always a combination of those from alive and dead cells, and are also formed by biogeochemical factors shaping microbial communities over decades. Therefore, they show the legacy of long-term changes in the microbial community. Because of the long duration of our experiment, it is possible that potential changes in the microbiome due to the year-long cessation of N-addition are overruled by the legacy of long-term eutrophication and warming.

## **Supplementary figures**


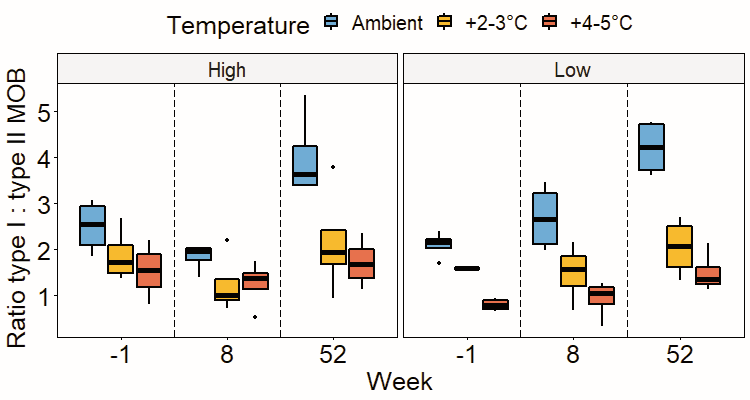


**Supplementary figure S1.** Ratio between type I and type II MOB in relation to temperature, nutrients and N-addition.


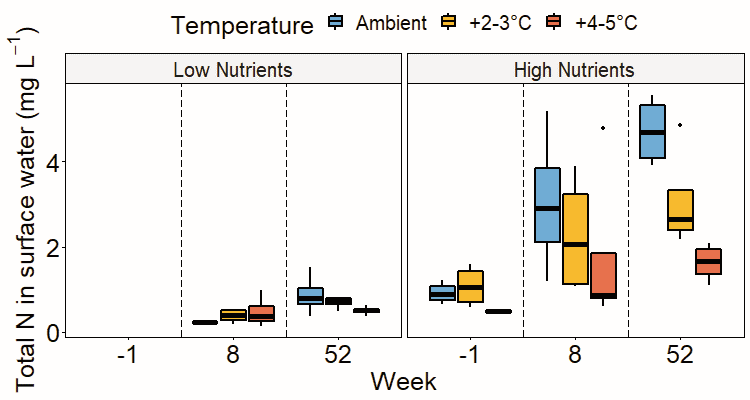


**Supplementary figure S2**. Total N in surface water in relation to temperature, nutrients and N-addition. No data for week -1 in low nutrients mesocosms.


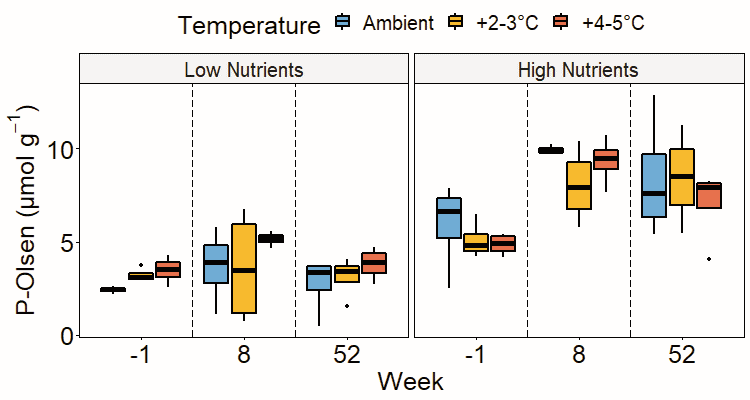


**Supplementary figure S3**. P-Olsen in top 4 cm of the sediment in relation to temperature, nutrients and N-addition.


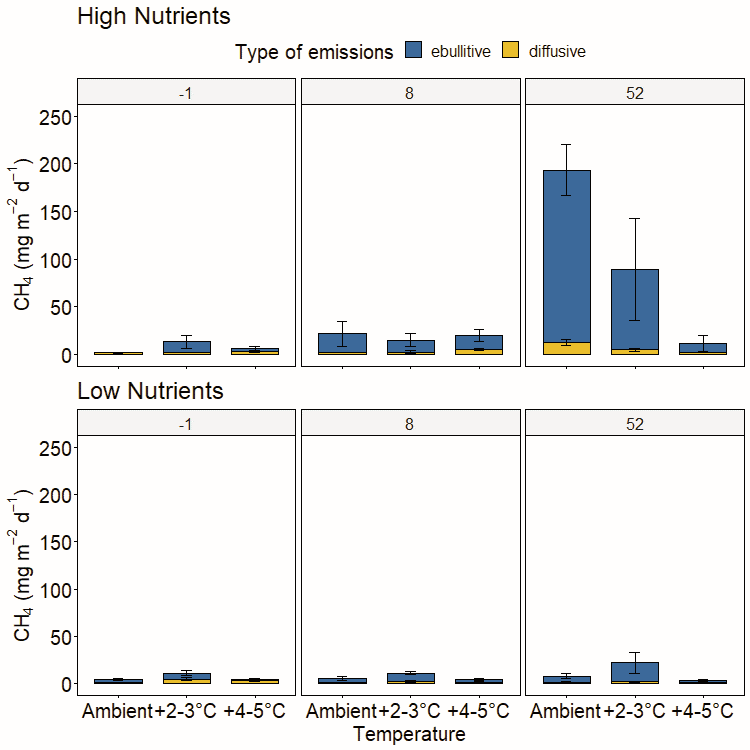


**Supplementary figure S4**. Diffusive and ebullitive CH_4_ emissions from the lake mesocosms in relation to temperature, nutrients and N-addition.


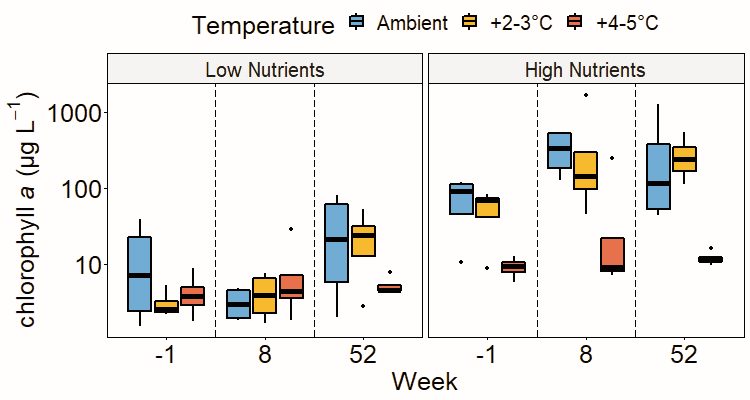


**Supplementary figure S5**. Chlorophyll *a* content (µg/l) in lake mesocosm surface waters in relation to temperature, nutrients and N-addition, shown in log scale.


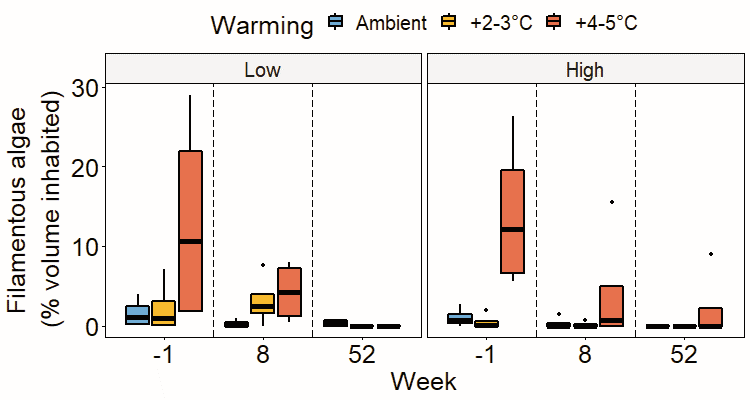


**Supplementary figure S6**. Percentage volume inhabited by filamentous algae in relation to temperature, nutrients and N-addition.


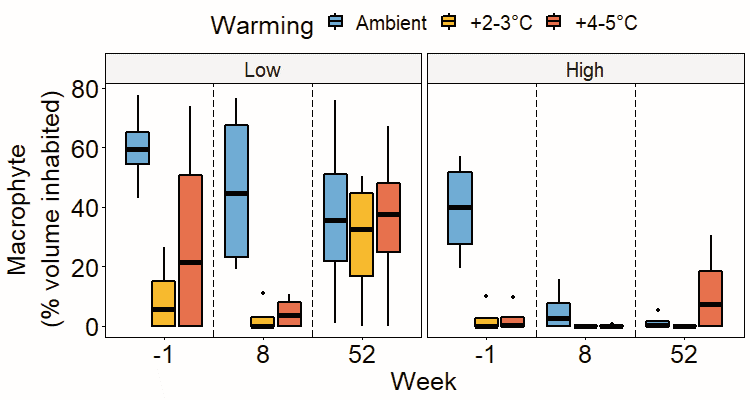


**Supplementary figure S7**. Percentage volume inhabited by macrophytes in the lake mesocosms in relation to temperature, nutrients and N-addition.


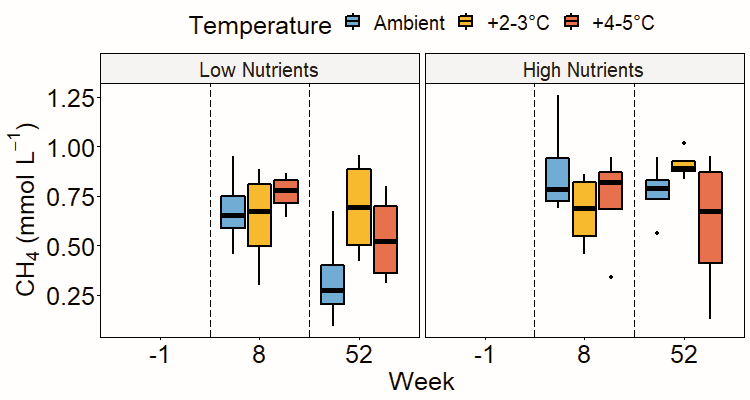


**Supplementary figure S8.** CH_4_ concentration in the pore water in relation to temperature, nutrients and N-addition.


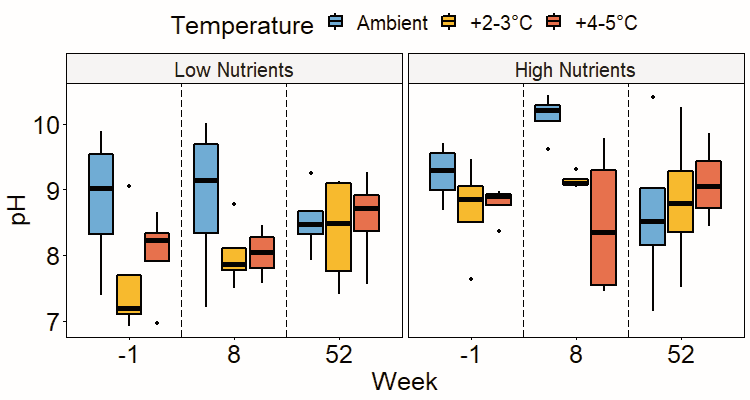


**Supplementary figure S9**. pH in the mesocosms, in relation to temperature, nutrients and N-addition, as measured with Hach pH sensor.


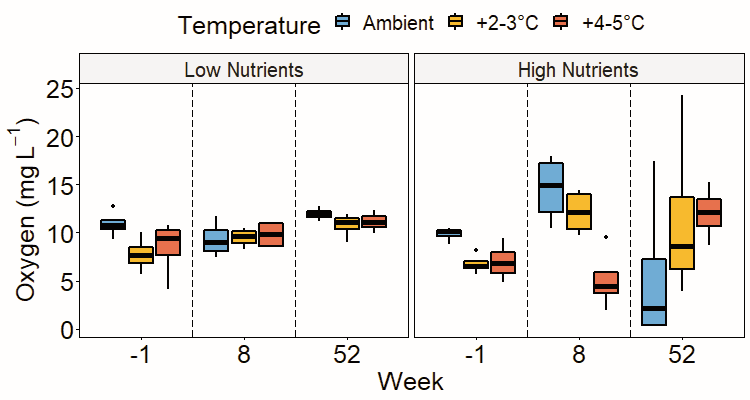


**Supplementary figure S10**. Oxygen concentration in the water column at 50 cm depth, in relation to temperature, nutrients and N-addition, based on the daily average of half hourly measurements.


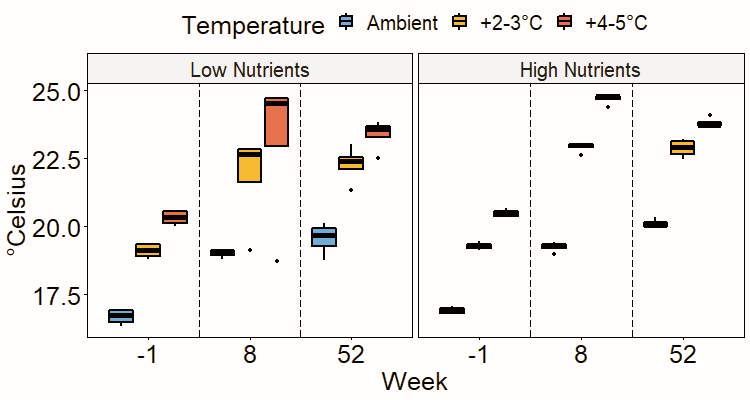


**Supplementary figure S11**. Actual temperature in the mesocosms at 50 cm depth, in relation to temperature, nutrients and N-addition, based on the daily average of half hourly measurements.

## **Supplementary tables**

**Supplementary table S1**: Three-way mixed ANOVA **(A)** and post-hoc Bonferroni adjusted t-tests **(B)** for square-root transformed MOB abundance in relation to temperature, nutrients and week (before/after resuming N-addition).

**A**

| Effect | Within / between subject | DFn | DFd | F | p | sign. |
| --- | --- | --- | --- | --- | --- | --- |
| Temp | Between | 2 | 18 | 11.4 | 0.0006 | *** |
| Nutr | Between | 1 | 18 | 6.8 | 0.018 | * |
| Week | Within | 2 | 36 | 3.4 | 0.044 | * |
| Temp:Nutr | - | 2 | 18 | 0.8 | 0.466 | ns |
| Temp:Week | - | 4 | 36 | 1.2 | 0.343 | ns |
| Nutr:Week | - | 2 | 36 | 2.1 | 0.133 | ns |
| Temp:Nutr:Week | - | 4 | 36 | 0.5 | 0.743 | ns |

**B**

| Effect | Tested on | Group1 | Group2 | T | df | p.adjusted | sign. |
| --- | --- | --- | --- | --- | --- | --- | --- |
| Temp | Whole dataset | Ambient | +2-3 °C | -6.62 | 23 | 2.82E-06 | **** |
| Temp | Whole dataset | Ambient | +4-5 °C | -6.44 | 23 | 4.32E-06 | **** |
| Temp | Whole dataset | +2-3 °C | +4-5 °C | -0.80 | 23 | 1.00 | ns |
| Week | Only high nutrient tanks | 0 | 8 | -3.61 | 11 | 0.012 | * |
| Week | Only high nutrient tanks | 0 | 52 | -1.51 | 11 | 0.477 | ns |
| Week | Only high nutrient tanks | 8 | 52 | 1.63 | 11 | 0.393 | ns |

**Supplementary table S2**: Three-way mixed ANOVA **(A)** and post-hoc Bonferroni adjusted t-tests **(B)** for square-root transformed methane oxidation potential in relation to temperature, nutrients and week (before/after resuming N-addition).

**A**

| Effect | Within / between subject | DFn | DFd | F | p | sign. |
| --- | --- | --- | --- | --- | --- | --- |
| Temp | Between | 2 | 17 | 1 | 0.292 | ns |
| Nutr | Between | 1 | 17 | 10 | 0.002 | *** |
| Week | Within | 2 | 34 | 30 | <0.0001 | **** |
| Temp:Nutr | - | 2 | 17 | 0.7 | 0.52 | ns |
| Temp:Week | - | 4 | 34 | 1 | 0.315 | ns |
| Nutr:Week | - | 2 | 34 | 2 | 0.103 | ns |
| Temp:Nutr:Week | - | 4 | 34 | 0.2 | 0.945 | ns |

**B**

| Effect | Tested on | Group1 | Group2 | T | df | p.adjusted | sign. |
| --- | --- | --- | --- | --- | --- | --- | --- |
| Week | Only high nutrient tanks | 0 | 8 | -10.68 | 11 | 1.15E-06 | **** |
| Week | Only high nutrient tanks | 0 | 52 | -6.32 | 11 | 1.70E-04 | *** |
| Week | Only high nutrient tanks | 8 | 52 | 2.01 | 11 | 2.10E-01 | ns |

**Supplementary table S3**: Three-way mixed ANOVA **(A)** and post-hoc Bonferroni adjusted t-tests **(B)** for the log-transformed MOB specific CH_4_ oxidation rate in relation to temperature, nutrients and week (before/after resuming N-addition).

**A**

| Effect | Within / between subject | DFn | DFd | F | p | sign. |
| --- | --- | --- | --- | --- | --- | --- |
| Temp | Between | 2 | 17 | 21.647 | 2.12E-05 | **** |
| Nutr | Between | 1 | 17 | 1.231 | 0.283 | ns |
| Week | Within | 2 | 34 | 25.229 | 1.92E-07 | **** |
| Temp:Nutr | - | 2 | 17 | 1.054 | 0.37 | ns |
| Temp:Week | - | 4 | 34 | 1.083 | 0.38 | ns |
| Nutr:Week | - | 2 | 34 | 1.943 | 0.159 | ns |
| Temp:Nutr:Week | - | 4 | 34 | 0.733 | 0.576 | ns |

**B**

| Effect | Tested on | Group1 | Group2 | T | df | p.adjusted | sign. |
| --- | --- | --- | --- | --- | --- | --- | --- |
| Temp | Whole dataset | Ambient | +2-3 °C | 4.78 | 23 | 0.0002 | *** |
| Temp | Whole dataset | Ambient | +4-5 °C | 8.60 | 23 | 5.22E-08 | **** |
| Temp | Whole dataset | +2-3 °C | +4-5 °C | 3.86 | 23 | 0.003 | ** |
| Week | Only high nutrient tanks | 0 | 8 | -4.13 | 11 | 0.005 | ** |
| Week | Only high nutrient tanks | 0 | 52 | -2.68 | 11 | 0.064 | ns |
| Week | Only high nutrient tanks | 8 | 52 | 0.19 | 11 | 1 | ns |

**Supplementary table S4**: PERMANOVA of main effects **(A)** and pairwise comparisons of warming treatments per nutrient level **(B)** related to MOB community composition. Monte-Carlo adjusted p-values used to determine significance. Mesocosm nested in nutrient and temperature treatments as random factor. Nperm=99999.

**A**

| Source | df | SS | MS | Pseudo-F | P(perm) | perms | P (Monte Carlo) | Sign. |
| --- | --- | --- | --- | --- | --- | --- | --- | --- |
| Nutr | 1 | 2441.9 | 2441.9 | 3.8308 | 0.0012 | 93924 | 0.001 | **** |
| Temp | 2 | 10813 | 5406.4 | 8.4814 | 1.00E-05 | 93884 | 1.00E-05 | **** |
| Week | 2 | 3699.3 | 1849.6 | 7.0465 | 1.00E-05 | 92684 | 1.00E-05 | **** |
| Temp:Nutr | 2 | 1013.2 | 506.62 | 0.79476 | 0.7027 | 92143 | 0.7023 | Ns |
| Nutr:Week | 2 | 1006.3 | 503.17 | 1.9169 | 0.0105 | 91916 | 0.0163 | * |
| Temp:Week | 4 | 1472.7 | 368.17 | 1.4026 | 0.0656 | 89691 | 0.0789 | Ns |
| Meso(Nutr:Temp) | 18 | 11474 | 637.44 | 2.4284 | 1.00E-05 | 82439 | 1.00E-05 | **** |
| Temp:Nutr:Week | 4 | 785.59 | 196.4 | 0.74821 | 0.8698 | 89273 | 0.8541 | ns |
| Res | 36 | 9449.7 | 262.49 |  |  |  |  |  |
| Total | 71 | 42155 |  |  |  |  |  |  |

**B**

| Nutrients | Group1 | Group2 | t | P(perm) | unique perms | P (Monte Carlo) | Sign. |
| --- | --- | --- | --- | --- | --- | --- | --- |
| Low | Ambient | +2-3 °C | 1.8822 | 0.0275 | 35 | 0.016 | * |
| Low | Ambient | +4-5 °C | 2.3238 | 0.0288 | 35 | 0.002 | ** |
| Low | +2-3 °C | +4-5 °C | 0.93709 | 0.4306 | 35 | 0.5128 | ns |
| High | Ambient | +2-3 °C | 2.2386 | 0.029 | 35 | 0.0038 | ** |
| High | Ambient | +4-5 °C | 3.4664 | 0.0273 | 35 | 0.0002 | *** |
| High | +2-3 °C | +4-5 °C | 1.6066 | 0.0859 | 35 | 0.0456 | * |

**Supplementary table S5**: relative abundance of different MOB genera and families in relation to temperature, nutrients and week (before/after resuming N-addition).

| MOB | Low Nutrients | | | High Nutrients | | |
| --- | --- | --- | --- | --- | --- | --- |
| Type 1 | Ambient | +2-3 °C | +4-5 °C | Ambient | +2-3 °C | +4-5 °C |
| *Methylomonadaceae* | 53.6 | 42.7 | 28.7 | 51.4 | 38.2 | 33.1 |
| *Methyloparacoccus* | 8.8 | 8.5 | 10.4 | 11.6 | 12.8 | 15.6 |
| *Methylocaldum* | 2.5 | 3.2 | 4.1 | 2.3 | 3.3 | 3.5 |
| *Methylobacter* | 3.2 | 3.1 | 2.3 | 3.0 | 3.0 | 2.2 |
| *Methylococcaceae* | 3.7 | 2.7 | 3.1 | 2.2 | 2.4 | 2.6 |
| *Methylovulum* | 1.6 | 1.6 | 1.2 | 1.2 | 1.0 | 1.0 |
| Total | 73.4 | 61.8 | 49.9 | 71.6 | 60.7 | 58.1 |
| Type 2 |  |  |  |  |  |  |
| *Methylocystis* | 24.6 | 34.0 | 44.9 | 26.2 | 37.1 | 37.5 |
| *Methylosinus* | 0.5 | 1.5 | 1.9 | 0.8 | 0.8 | 1.9 |
| *Methyloceanibacter* | 0.4 | 1.5 | 2.1 | 1.0 | 0.8 | 1.9 |
| Other Alphaproteob. | 1.0 | 1.3 | 1.2 | 0.4 | 0.6 | 0.7 |
| Total type 2 | 26.6 | 38.2 | 50.1 | 28.4 | 39.3 | 41.9 |

**Supplementary table S6**: Three-way mixed ANOVA **(A)** and post-hoc Bonferroni adjusted t-tests **(B)** for the log-transformed ratio type I: type II MOB in relation to temperature, nutrients and week (before/after resuming N-addition).

**A**

| Effect | Within / between subject | DFn | DFd | F | p | sign. |
| --- | --- | --- | --- | --- | --- | --- |
| Temp | Between | 2 | 18 | 17.432 | 6.15E-05 | **** |
| Nutr | Between | 1 | 18 | 0.422 | 0.524 | ns |
| Week | Within | 2 | 36 | 24.497 | 1.92E-07 | **** |
| Temp:Nutr | - | 2 | 18 | 1.346 | 0.285 | ns |
| Temp:Week | - | 4 | 36 | 1.668 | 0.179 | ns |
| Nutr:Week | - | 2 | 36 | 3.254 | 0.05 | ns |
| Temp:Nutr:Week | - | 4 | 36 | 0.568 | 0.688 | ns |

**B**

| Effect | Tested on | Group1 | Group2 | T | df | p.adjusted | sign. |
| --- | --- | --- | --- | --- | --- | --- | --- |
| Temp | Whole dataset | Ambient | +2-3 °C | 5.64 | 23 | 2.91E-05 | **** |
| Temp | Whole dataset | Ambient | +4-5 °C | 7.43 | 23 | 4.50E-07 | **** |
| Temp | Whole dataset | +2-3 °C | +4-5 °C | 3.22 | 23 | 0.011 | * |
| Week | Only high nutrient tanks | 0 | 8 | 2.90 | 11 | 0.044 | * |
| Week | Only high nutrient tanks | 0 | 52 | -2.42 | 11 | 0.102 | ns |
| Week | Only high nutrient tanks | 8 | 52 | -5.70 | 11 | 0.0004 | *** |

**Table S7**: BLAST search results for all *Crenothrix* OTU’s with over 0.5% abundance

| Silva  Family | Silva  Genus | ASV (numbers in order of rel. abu. in complete 16S dataset) | Relative abundance | First identified hit BLAST | % identity | Accession | *Methylobacter* hits | *Crenothrix* hits | RDP taxonomy assigned |
| --- | --- | --- | --- | --- | --- | --- | --- | --- | --- |
| *Methylomonadaceae* | *Crenothrix* | 87 | 9.34% | Uncultured *Methylobacter* sp. clone BWET1cm53 | 100 | [JQ793253.1](https://www.ncbi.nlm.nih.gov/nucleotide/JQ793253.1?report=genbank&log$=nucltop&blast_rank=4&RID=97HPY00W016) | 16 of 100 | 1 hit, uncultured clone, KX366045.1, 99.12% similarity | *Methylobacter* |
| *Methylomonadaceae* | *Crenothrix* | 165 | 5.62% | Uncultured *Methylobacter* sp. clone Xh_Meth1b_CA18 | 100 | [JQ038191.1](https://www.ncbi.nlm.nih.gov/nucleotide/JQ038191.1?report=genbank&log$=nucltop&blast_rank=4&RID=97HPY00W016) | 17 of 100 | 0 in first 100 hits | *Methylobacter* |
| *Methylomonadaceae* | *Crenothrix* | 185 | 5.41% | Uncultured Methylobacter sp. clone BWET1cm53 | 99.56 | [JQ793253.1](https://www.ncbi.nlm.nih.gov/nucleotide/JQ793253.1?report=genbank&log$=nucltop&blast_rank=4&RID=97HPY00W016) | 14 of 100 | 1 hit, uncultured clone, KX366045.1, 98.68% similarity | *Methylobacter* |
| *Methylomonadaceae* | *Crenothrix* | 189 | 5.37% | Uncultured *Methylobacter* sp. clone Xh_Meth1b_TO20 | 100 | [JQ038189.1](https://www.ncbi.nlm.nih.gov/nucleotide/JQ038189.1?report=genbank&log$=nucltop&blast_rank=1&RID=97HPY00W016) | 30 of 100 | 0 in first 100 hits | *Methylobacter* |
| *Methylomonadaceae* | *Crenothrix* | 207 | 4.35% | Uncultured *Methylobacter* sp. clone BWET1cm53 | 99.12 | [JQ793253.1](https://www.ncbi.nlm.nih.gov/nucleotide/JQ793253.1?report=genbank&log$=nucltop&blast_rank=4&RID=97HPY00W016) | 14 of 100 | 1 hit, uncultured clone, KX366045.1, 98.24% similarity | *Methylobacter* |
| *Methylomonadaceae* | *Crenothrix* | 275 | 3.50% | Uncultured *Methylobacter* sp. clone Xh_Meth1b_TO20 | 99.56 | [JQ038189.1](https://www.ncbi.nlm.nih.gov/nucleotide/JQ038189.1?report=genbank&log$=nucltop&blast_rank=1&RID=97HPY00W016) | 30 of 100 | 0 in first 100 hits | *Methylobacter* |
| *Methylomonadaceae* | *Crenothrix* | 642 | 1.36% | Uncultured *Methylobacter* sp. clone BWET3cm92 | 100 | [JQ793373.1](https://www.ncbi.nlm.nih.gov/nucleotide/JQ793373.1?report=genbank&log$=nucltop&blast_rank=5&RID=97HPY00W016) | 20 of 100 | 1 hit, uncultured clone, KX366045.1, 99.11% similarity | *Methylobacter* |
| *Methylomonadaceae* | *Crenothrix* | 782 | 1.04% | Uncultured *Methylobacter* sp. clone Xh_Typel_PA6 | 100 | [JQ038182.1](https://www.ncbi.nlm.nih.gov/nucleotide/JQ038182.1?report=genbank&log$=nucltop&blast_rank=4&RID=97HPY00W016) | 16 of 100 | 0 in first 100 hits | *Methylobacter* |
| *Methylomonadaceae* | *Crenothrix* | 983 | 0.85% | Uncultured *Methylobacter* sp. clone Xh_Meth1b_TO20 | 99.56 | [JQ038189.1](https://www.ncbi.nlm.nih.gov/nucleotide/JQ038189.1?report=genbank&log$=nucltop&blast_rank=1&RID=97HPY00W016) | 14 of 100 | 0 in first 100 hits | *Methylobacter* |

## **Supplementary references**

1. Liboriussen L et al. Global warming: Design of a flow-through shallow lake mesocosm climate experiment. *Limnol Oceanogr Methods* 2005; **3**: 1–9.

2. Cooper R, McCarthy J, Metz B. Climate Change 2001: The Scientific Basis. *Foreign Aff* 2002; **81**: 208.

3. de Jong AEE et al. Increases in temperature and nutrient availability positively affect methane-cycling microorganisms in Arctic thermokarst lake sediments. *Environ Microbiol* 2018; **20**: 4314–4327.

4. Costello AM, Lidstrom ME. Molecular characterization of functional and phylogenetic genes from natural populations of methanotrophs in lake sediments. *Appl Environ Microbiol* 1999; **65**: 5066–5074.

5. Sundberg C et al. 454 pyrosequencing analyses of bacterial and archaeal richness in 21 full-scale biogas digesters. *FEMS Microbiol Ecol* 2013; **85**: 612–626.

6. Callahan BJ et al. DADA2: High-resolution sample inference from Illumina amplicon data. *Nat Methods* 2016; **13**: 581–583.

7. Quast C et al. The SILVA ribosomal RNA gene database project: Improved data processing and web-based tools. *Nucleic Acids Res* 2013; **41**: D590-596.

8. Weiss S et al. Normalization and microbial differential abundance strategies depend upon data characteristics. *Microbiome* 2017; **5**: 27.

9. McMurdie PJ, Holmes S. Phyloseq: An R Package for Reproducible Interactive Analysis and Graphics of Microbiome Census Data. *PLoS One* 2013; **8**: e61217.

10. Leo L, Shetty S. microbiome R package. *Bioconductor* . 2017.

11. Stoecker K et al. Cohn’s Crenothrix is a filamentous methane oxidizer with an unusual methane monooxygenase. *Proc Natl Acad Sci U S A* 2006; **103**: 2363–2367.

12. Oswald K et al. Crenothrix are major methane consumers in stratified lakes. *ISME J* 2017; **11**: 2124–2140.

13. Altschul SF, Gish W, Miller W, Myers EW, Lipman DJ. Basic Local Alignment Tool. *J Mol Biol* . 1990. , **215**: 403–410

14. Cole JR et al. Ribosomal Database Project: Data and tools for high throughput rRNA analysis. *Nucleic Acids Res* 2014; **42**: D633-42.

15. Olsen SR, Cole C V, Watandbe F, Dean L. Estimation of Available Phosphorus in Soil by Extraction with sodium Bicarbonate. *USDA Circular Nr 939* . 1954. US Government Printing Office, Washington, D.C.

16. FOSS-PO4-P. Determination of total phosphorus in water by FIAstar 5000, Application Note 5241 Acc to ISO 15681-1 Rev 4. *Co Man* 2008; **Applicatio**.

17. Danish Standards Foundation. Vandundersøgelse. Klorofyl a - spektrofotometrisk måling i ethanolekstrakt. *Dansk Stand* 1986; **DS 2201**: 1–6.

18. Almeida RM et al. High primary production contrasts with intense carbon emission in a eutrophic tropical reservoir. *Front Microbiol* 2016; **7**: 717.

19. Wik M, Crill PM, Varner RK, Bastviken D. Multiyear measurements of ebullitive methane flux from three subarctic lakes. *J Geophys Res Biogeosciences* 2013; **118**: 1307–1321.

20. DelSontro T, Boutet L, St-Pierre A, del Giorgio PA, Prairie YT. Methane ebullition and diffusion from northern ponds and lakes regulated by the interaction between temperature and system productivity. *Limnol Oceanogr* 2016; **61**: S62–S77.

21. Canfield DE et al. Prediction of chlorophyll a concentrations in Florida lakes: importance of aquatic macrophytes. *Can J Fish Aquat Sci* 1984; **41**: 497–501.

22. Kassambara A. Package ‘rstatix’: Pipe-Friendly Framework for Basic Statistical Tests. *R Packag version 060* . 2020.

23. Vallenet D et al. MicroScope - An integrated microbial resource for the curation and comparative analysis of genomic and metabolic data. *Nucleic Acids Res* 2013; **41**: 636–647.

24. Ghashghavi M, Jetten MSM, Lüke C. Survey of methanotrophic diversity in various ecosystems by degenerate methane monooxygenase gene primers. *AMB Express* 2017; **7**: 162.

25. Legendre P, Gallagher ED. Ecologically meaningful transformations for ordination of species data. *Oecologia* 2001; **129**: 271–280.

26. Oksanen J et al. Vegan: Community Ecology Package. R package version 2.0-9. *Community Ecol Packag* . 2013.

27. Holland SM. Non Metric Multidimensional Scaling (Nms). *Dep Geol Univ Georg Athens, Tech Rep GA* 2008; 30602–2501.

28. Anderson MJ. Permutational Multivariate Analysis of Variance (PERMANOVA). *Wiley StatsRef: Statistics Reference Online*. 2017. pp 1–15.

29. Anderson MJ, Gorley RN, Clarke KR. PERMANOVA+ for PRIMER: Guide to Software and Statistical Methods. *Plymouth, UK*. 2008. pp 1–214.

30. Valero-Mora PM. ggplot2: Elegant Graphics for Data Analysis. *J Stat Softw* 2010; **35**.

31. Kassambara A. Package ‘ggpubr’: ‘ggplot2’ Based Publication Ready Plots. *R Packag version 040* . 2020.

32. R Core Team. R: A language and environment for statistical computing. *R Found Stat Comput* . 2019.

33. Davidson TA et al. Synergy between nutrients and warming enhances methane ebullition from experimental lakes. *Nat Clim Chang* 2018; **8**: 156–160.
